# MAPLE-PD trial (Mesenteric Approach vs. Conventional Approach for Pancreatic Cancer during Pancreaticoduodenectomy): study protocol for a multicenter randomized controlled trial of 354 patients with pancreatic ductal adenocarcinoma

**DOI:** 10.1186/s13063-018-3002-z

**Published:** 2018-11-08

**Authors:** Seiko Hirono, Manabu Kawai, Ken-ichi Okada, Tsutomu Fujii, Masayuki Sho, Sohei Satoi, Ryosuke Amano, Hidetoshi Eguchi, Yuko Mataki, Masafumi Nakamura, Ippei Matsumoto, Hideo Baba, Masaji Tani, Yasunari Kawabata, Yuichi Nagakawa, Suguru Yamada, Yoshiaki Murakami, Toshio Shimokawa, Hiroki Yamaue

**Affiliations:** 1 Second Department of Surgery, Wakayama Medical University, School of Medicine, 811-1 Kimiidera, Wakayama, 641-8510 Japan; 2 Department of Surgery and Science, Graduate School of Medicine and Pharmaceutical Sciences, University of Toyama, Toyama, Japan; 3 Department of Surgery, Nara Medical University, Kashihara, Japan; 4 Department of Surgery, Kansai Medical University, Hirakata, Japan; 5 Department of Surgical Oncology, Osaka City University, Graduate School of Medicine, Osaka, Japan; 6 Department of Gastroenterological Surgery, Graduate School of Medicine, Osaka University, Osaka, Japan; 7 Department of Digestive Surgery, Breast and Thyroid Surgery, Kagoshima University, Kagoshima, Japan; 8 Department of Surgery and Oncology, Graduate School of Medical Sciences, Kyushu University, Fukuoka, Japan; 9 Department of Surgery, Kindai University Faculty of Medicine, Higashiosaka City, Japan; 10 Department of Gastroenterological Surgery, Graduate School of Life Science, Kumamoto University, Kumamoto, Japan; 11 Department of Surgery, Shiga University of Medical Science, Ōtsu, Japan; 12 Department of Digestive and General Surgery, Shimane University Faculty of Medicine, Izumo City, Japan; 13 Department of Gastrointestinal and Pediatric Surgery, Tokyo Medical University, Tokyo, Japan; 14 Department of Gastroenterological Surgery, Nagoya University, Graduate School of Medicine, Nagoya, Japan; 15 Department of Surgery, Institute of Biomedical and Health Science, Hiroshima University, Hiroshima, Japan; 16 Clinical Study Support Center, Wakayama Medical University, School of Medicine, Wakayama, Japan

**Keywords:** Mesenteric approach, Artery-first approach, Conventional approach, Pancreaticoduodenectomy, R0 resection, Pancreatic ductal adenocarcinoma, Resectable pancreatic cancer, Borderline resectable pancreatic cancer, Superior mesenteric artery

## Abstract

**Background:**

The mesenteric approach is an artery-first approach to pancreaticoduodenectomy for pancreatic cancer, which starts with the dissection of connective tissues around the superior mesenteric artery. The procedure aims for early confirmation of resectability by checking the surgical margin around the superior mesenteric artery first during the operation. It also aims to decrease intraoperative blood loss by early ligation of the inferior pancreaticoduodenal artery and to increase R0 rate by complete clearance of the lymph nodes around the superior mesenteric artery and pancreatic head plexus II, the most favorable positive margin site for pancreatic ductal adenocarcinoma. Furthermore, it aims to avoid the spread of cancer cells during operation (nontouch isolation technique). The MAPLE-PD (Mesenteric Approach vs. Conventional Approach for Pancreatic Cancer during Pancreaticoduodenectomy) trial investigates whether the mesenteric approach can prolong the survival of patients with pancreatic ductal adenocarcinoma who undergo pancreaticoduodenectomy compared with the conventional approach.

**Methods/design:**

The MAPLE-PD trial is a Japanese multicenter randomized controlled trial that compares the surgical outcomes between the mesenteric and conventional approaches to pancreaticoduodenectomy. Patients with pancreatic ductal adenocarcinoma scheduled to undergo pancreaticoduodenectomy are randomized before operation to either a conventional approach (arm A) or a mesenteric approach (arm B). In arm A, the operation starts with Kocher’s maneuver. At the final step of the removal procedure, the connective tissues around the superior mesenteric artery are dissected. In arm B, the operation starts with dissection of the connective tissues around the superior mesenteric artery and ends with Kocher’s maneuver. In total, 354 patients from 15 Japanese high-volume centers will be randomized. The primary endpoint is overall survival by intention-to-treat analysis. Secondary endpoints include intraoperative blood loss, R0 rate, and recurrence-free survival.

**Discussion:**

If the MAPLE-PD trial shows the oncological benefits of the mesenteric approach for patients with pancreatic ductal adenocarcinoma, this procedure may become a standard approach to pancreaticoduodenectomy.

**Trial registration:**

ClinicalTrials.gov, NCT03317886. Registered on 23 October 2017.

University Hospital Medical Information Network Clinical Trials Registry, UMIN000029615. Registered on 15 January 2018.

**Electronic supplementary material:**

The online version of this article (10.1186/s13063-018-3002-z) contains supplementary material, which is available to authorized users.

## Background

The only curative treatment for pancreatic ductal adenocarcinoma (PDAC) is currently complete surgical resection with adjuvant therapies. Pancreaticoduodenectomy (PD) is commonly accepted as a surgical treatment for PDAC located in the pancreatic head. Because the pathological positive margins (R1) of the resected specimen can cause postoperative recurrence and poor survival, R0 resection is necessary to improve the survival of patients with PDAC [1–3]. The dissected margins around the superior mesenteric artery (SMA) are reported to be the most favorable R1 site for PDAC in the pancreatic head [3, 4]. This region includes the lymph nodes, the inferior pancreaticoduodenal artery (IPDA), and the nerve plexus from the SMA to the pancreatic head, which is defined as the pancreatic head plexus II (plPh-II) in the fourth English edition of the classification of pancreatic carcinoma organized by the Japanese Pancreatic Society [5].

PD generally starts with Kocher’s maneuver, including exfoliation of the pancreatic head and the duodenum from the retroperitoneum, followed by regional lymphadenectomy and division of the stomach, bile duct, and pancreas. Finally, dissection of the connective tissue around the SMA, including the lymph nodes, plPh-II, and IPDA, is performed before removal of the specimen. We refer to this typical procedure as the conventional approach [6].

Recently, the use of the term *artery-first approach* during PD has spread worldwide [7, 8]. The concept of the artery-first approach is to start by dissection of the connective tissues around the SMA during PD. The aims are (1) early determination of the resectability status before taking any irreversible step by checking the degree of cancer invasion to the SMA plexus macroscopically and microscopically, which is considered to be the most favorable surgical positive margin; and (2) to decrease intraoperative blood loss by early control of blood inflow into the pancreatic head by division of the IPDA in an early stage of PD [8].

The mesenteric approach, first suggested by Nakao et al. in 1993 [9], is an artery-first approach during PD. The mesenteric approach starts with complete clearance of the connective tissues around the superior mesenteric vein (SMV) and the SMA from the infracolic mesenterium to the mesenteric root [9, 10]. After regional lymphadenectomy and division of the stomach, bile duct, and pancreas, which are performed in the same way as in the conventional approach, exfoliation of the pancreatic head and duodenum from the retroperitoneum (Kocher’s maneuver) is the final step before removal of the specimen. The mesenteric approach allows dissection around the SMA from the noncancerous and less inflammatory side of the mesenterium, and it may therefore be considered a safer procedure than other approaches to PD by preventing cancer cell spread during operation by a nontouch isolation technique and increasing the R0 rate by complete dissection of the connective tissues around the SMA and SMV, including the lymph nodes and the plPh-II [9, 10].

Our retrospective study first showed the surgical and oncological benefits of the mesenteric approach for patients with PDAC compared with the conventional approach [6]. To confirm the results of our retrospective study and to evaluate the clinical benefits of the mesenteric approach for patients with PDAC, we are conducting a randomized, controlled, multicenter trial comparing the oncological and surgical outcomes between the mesenteric and conventional approaches during PD for patients with PDAC (Mesenteric Approach vs. Conventional Approach for Pancreatic Cancer during Pancreaticoduodenectomy [MAPLE-PD] trial).

## Methods/design

### Design

The MAPLE-PD trial is a Japanese multicenter, randomized, controlled trial. Patients with PDAC are randomized to arm A (conventional approach) or arm B (mesenteric approach) during PD. The MAPLE-PD trial is conducted in 15 Japanese high-volume centers (Additional file 1, institution list) that have been board-certified as training institutions by the Japanese Society of Hepato-Biliary-Pancreatic Surgery, and the interventions are performed by instructors and expert surgeons certified by the Japanese Society of Hepato-Biliary-Pancreatic Surgery to ensure the high quality of the study. The aim of this study is to evaluate the oncological and surgical benefits of the mesenteric approach during PD for patients with PDAC compared with the conventional approach. Therefore, this study is designed to evaluate the superiority of the mesenteric approach (arm B) compared with the conventional approach (arm A) during PD in terms of overall survival (OS).

**Fig. 1:**
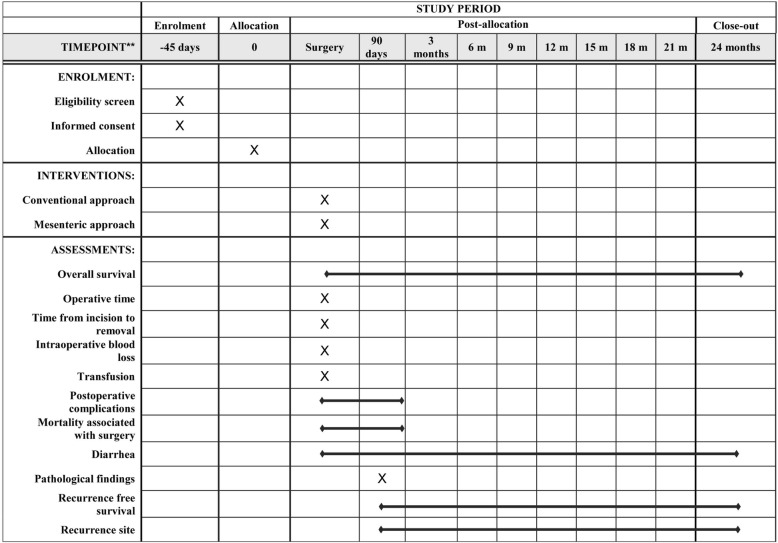
Study calendar

All patients are required to undergo postoperative examination every 3 months for at least 2 years after surgery. Signs of suspected recurrence of disease will be closely monitored. The schedule of this trial is shown in Fig. 1. The study period of the MAPLE-PD trial is expected to be 4 years, including 2 years for patient recruitment and 2 years for follow-up.

### Study endpoints

The primary endpoint is OS. Secondary endpoints include operative time, time from skin incision to removal of the specimen, intraoperative blood loss volume, and transfusion. Further secondary endpoints include incidence of postoperative complications within 90 days after surgery, including grade B or C pancreatic fistula, delayed gastric emptying (DGE), and intra-abdominal hemorrhage; all-morbidity rate within 90 days after surgery; mortality rate within 90 days after surgery; and incidence of diarrhea. The rates of R0 and R1; the closest length from tumor margin to surgical margin in the case of R0 resection; number of harvested lymph nodes, number of metastatic lymph nodes, and lymph node ratio; recurrence-free survival; and site of initial recurrence are also secondary endpoints.

Postoperative pancreatic fistula (POPF) [11], DGE [12], and intra-abdominal hemorrhage [13] are defined and graded according to the International Study Group of Pancreatic Surgery (ISGPS). Postoperative complications other than POPF, DGE, and intra-abdominal hemorrhage are graded by Clavien-Dindo classification [14]. Postoperative diarrhea is graded according to the Common Terminology Criteria for Adverse Events version 4.

### Statistical analysis

#### Sample size

A retrospective study at the Wakayama Medical University Hospital showed that the 2-year OS rates were 28.5% for patients with PDAC treated by the conventional approach and 50.7% for those treated by the mesenteric approach [6]. The HR of this result was calculated to be 0.541. However, we considered the possibility that the HR of the MAPLE-PD trial would be higher than 0.541, because recent advanced perioperative management and adjuvant chemotherapy might improve survival of patients with PDAC treated by either approach. In the MAPLE-PD trial, we therefore set the HR at 0.70. To detect an HR of 0.70 with 80% power using a two-sided log-rank test at the 0.05 level of significance requires 134 events in the conventional approach group and 115 events in the mesenteric approach group. To observe the number of these events during the 4-year study period, 354 patients (177 patients in each group) are necessary (accrual time is 2 years and follow-up time is 2 years), considering a dropout proportion of 10% for each group.

#### Statistical analysis plan

The primary population for efficacy analysis will be the intention-to-treat population, defined as all randomized patients. The primary endpoint is OS. The null hypothesis that the HR is equal to 0.70, which will be tested against the alternative hypothesis that the HR is not equal to 0.70. The primary endpoint will be compared using a stratified log-rank test with a two-sided alpha of 0.05 stratified by resectability (resectable versus BR-PV), preoperative adjuvant therapy (yes or no), and the participating institution. Survival analysis will be conducted using the Kaplan-Meier survival curves and the log-rank test in the two randomization groups. The HRs and 95% CIs will be estimated by the Cox proportional hazards model.

For the secondary endpoints, categorical outcomes will be summarized using frequency and percentage for each arm and will be compared using Fisher’s exact method. Continuous outcomes will use median and range for each arm and will be compared using the Wilcoxon rank-sum test. Survival outcomes will be analyzed using the Kaplan-Meier method and compared by log-rank test.

### Study population

Patients are eligible if they meet the MAPLE-PD trial definitions for resectable PDAC or borderline resectable pancreatic ductal adenocarcinoma with portal vein invasion (BR-PV PDAC) and are scheduled to undergo PD. Resectable PDAC and BR-PV PDAC are defined according to the National Comprehensive Cancer Network (NCCN) definition 2017 [15].

#### Inclusion criteria

Eligible for enrollment in the study are patients who are scheduled to undergo elective PD for resectable PDAC or BR-PV PDAC, have an Eastern Cooperative Oncology Group performance status of 0–1, are ≥ 20 years old, have intact function of major organs (e.g., bone marrow, heart, liver, kidney, lung), and have provided written informed consent based on sufficient understanding of the study.

The exclusion criteria are as follows:

1. Patients with severe ischemic cardiovascular disease
2. Patients with liver cirrhosis or active hepatitis
3. Patients needing oxygen owing to interstitial pneumonia or lung fibrosis
4. Patients undergoing dialysis for chronic renal failure
5. Patients needing surrounding organ resection
6. Patients needing arterial reconstruction (e.g., SMA, common hepatic artery, celiac axis)
7. Patients with suspicious para-aortic lymph node metastases visualized by preoperative imaging
8. Patients having active multiple cancer that is thought to influence the occurrence of adverse events or survival
9. Patients receiving long-term steroid medication that is thought to influence the occurrence of adverse events
10. Patients scheduled to undergo laparoscopic or laparoscopy-assisted PD
11. Patients having difficulty with study participation owing to psychotic disease or symptoms
12. Patients whose preoperative biopsy tissues are diagnosed as pathological findings other than PDAC
13. Patients who have undergone gastrostomy or colon/rectum resection previously
14. Patients with severe drug allergy to iodine and gadolinium

Exit criteria from the protocol include the following:

1. Patients offering to cancel entry into the MAPLE-PD trial after enrollment
2. Patients who are found to have distant metastasis and/or peritoneal dissemination during surgery
3. Patients with macroscopic positive margins (R2)
4. Patients requiring change of treatment procedure, such as combined other organ resection bypass procedure and exploratory laparotomy
5. Patients needing to cancel the treatment procedure during surgery owing to intraoperative cardiac infarction, heavy bleeding, and so forth
6. Patients with pathological disease other than PDAC

#### Randomization

**Fig. 2:**
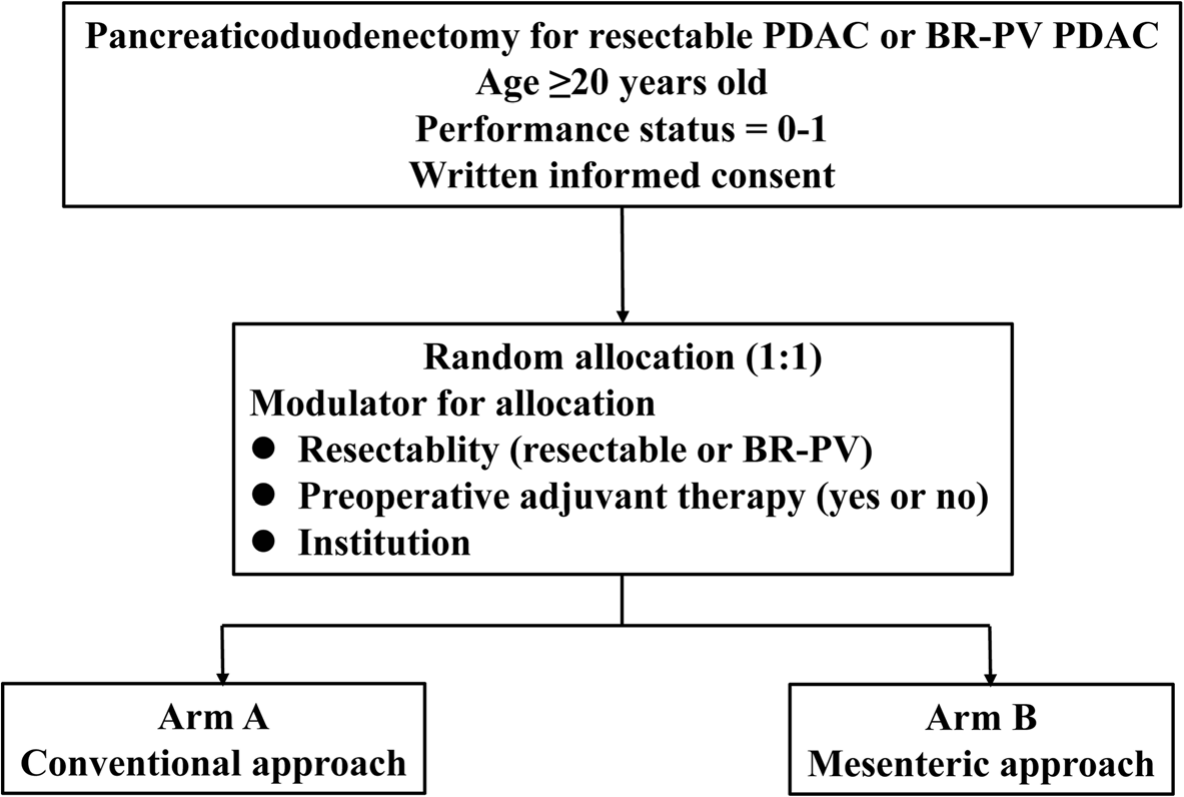
Flow diagram of the MAPLE-PD (Mesenteric Approach vs. Conventional Approach for Pancreatic Cancer During Pancreaticoduodenectomy) trial

After confirmation of eligibility, including written informed consent, patients are randomized in a 1:1 allocation ratio to either arm A (conventional approach) or arm B (mesenteric approach) with a random block size. Central randomization and registration will be applied, using an electronic data capture (EDC) system. After being assessed for eligibility at registration, patients will be centrally randomized to either arm A or arm B. To minimize background bias between the two groups, this study is stratified for resectability (resectable versus BR-PV), according to preoperative adjuvant therapy (yes or no), and by participating institution. The indication and regimen for preoperative adjuvant therapies depend on the treatment strategy of the participating institutions. We use Pocock and Simon’s minimization method for random assignment and the Mersenne Twister for random number generation (Fig. 2, flow diagram of the MAPLE-PD trial).

All patients are blinded to the surgical approach that they will be receiving, and they are required to sign an informed consent before enrolling in this study. Blinding of the surgeons is not possible, owing to the different techniques used during the operation. However, the result assessment will be made by an independent researcher (T.S.) who will be blinded to the surgical procedures.

### Interventions

#### Trial intervention (mesenteric approach)

After laparotomy, the mesenterium is incised at the line between the Treitz ligament and the inferior duodenal flexure to identify the SMA and the SMV. The middle colic artery is exposed arising from the anterior side of the SMA, and this artery is usually divided. The lymph node dissection around the SMA proceeds to the origin of the SMA in a longitudinal direction, and the IPDA or the common trunk of the IPDA and J1 artery is ligated and divided at the root. Lymph node dissection around the SMV is also performed. The mesenteric approach is completed when the circumferential dissection of the connective tissues around the SMA and the SMV, including lymph nodes (#14), plPh-II, and IPDA, is performed [9, 10, 16].

Regional lymph node dissection around the common hepatic artery (#8), the root of the left gastric artery (#7), the right side of the celiac axis (#9), and in the hepatoduodenal ligament (#12) is performed. After the stomach, bile duct, pancreas, and jejunum are divided, the tumor with en bloc-dissected tissues is removed. Finally, the pancreatic head is exfoliated from the retroperitoneum by Kocher’s maneuver. If tumor invasion of the portal vein (PV)/SMV is suspected pre- and/or intraoperatively, concomitant PV/SMV resection is performed immediately before the specimen is removed and reconstruction of PV/SMV is performed [17]. In reconstruction, pancreaticojejunostomy or pancreaticogastrostomy, choledochojejunostomy, and gastrojejunostomy are performed in turn.

#### Control intervention (conventional approach)

Following exfoliation of the pancreatic head and duodenum from the retroperitoneum by Kocher’s maneuver, regional lymph node dissection around the same areas as those of the mesenteric approach (#7, #8, #9, and #12), and the stomach or duodenum, bile duct, and pancreas are divided. Finally, dissection of the connective tissues around the SMA, including lymph node and plPh-II, and division of the IPDA are performed before removal of the specimen. After complete isolation of the pancreatic head from the SMA, the jejunum is transected, and the specimen is removed. The same reconstruction as that of the mesenteric approach is then performed.

### Standardization and validation of interventions

Before the start of the MAPLE-PD trial, we held three consensus meetings to determine the details of the operative techniques for both groups by observing and discussing several operative videos to ensure standardization of the interventions in all institutions. On the basis of these meetings, we determined that a photographic record after the mesenteric approach is necessary for the arm B group to validate intervention quality, although this is not necessary for the conventional approach (arm A), because the operative techniques of this approach have been certified by the Japanese Society of Hepato-Biliary-Pancreatic Surgery in all institutions participating in the MAPLE-PD trial. Central judgment will be conducted for the arm B group, and the photographs will be reviewed by more than two members of the committee at that time.

### Recruitment

To achieve adequate participant enrollment to reach the target sample size within the study period, 15 Japanese high-volume centers will participate in the MAPLE-PD trial.

### Follow-up

After randomization, the patients will be followed every 3 months, or more often if the patient’s situation requires, for at least 2 years. The patients in this study will undergo computed tomography or magnetic resonance imaging every 3 months to evaluate postoperative recurrence and metastases. OS is defined as the time from operation to the time of the last follow-up or death. Recurrence-free survival is defined as the time from operation to the time of finding any recurrence or metastasis or until death.

### Data and safety monitoring

An independent data monitoring committee (Clinical Study Support Center, Wakayama Medical University School of Medicine) will monitor the safety of the trial subjects by qualitative analyses of feasibility, accrual rate, and adverse events as well as dropouts every 6 months during the study. Data are collected via a case report form using an EDC system and paper and stored and managed securely by the data monitoring committee. After written consent is signed, at baseline, all baseline assessments will be conducted before randomization to improve the quality of data. The handling of all cases is managed by subject identification code or anonymized registration number. The correspondence table of the anonymizing codes and names, as well as consent form containing the name, is kept in separate restricted-access lockable document storage at each participating institution. To promote data quality, missing data will be pursued until received or confirmed as not available or until the trial reaches analysis.

No interim analyses are planned in the MAPLE-PD trial. The principal investigator has the right to terminate the trial at any time in consultation with the biostatistician. Reasons that may require termination of the trial include the following:

- The incidence or severity of adverse events in the trial indicates a potential health hazard caused by the study treatment
- It appears that the patient enrollment is unsatisfactory with respect to quality, or quantity or data recording is severely inaccurate or incomplete
- External evidence renders it necessary to terminate the trial

All adverse events observed by the investigators will be recorded up to 90 days after surgery and reported to the principal investigator and clinical trials coordination center. The assignment of the severity or grading should be made by the investigator responsible for the care of the participant. Serious adverse events are defined as those that are life-threatening or result in death. Serious adverse events will be collected and recorded according to good clinical practice throughout the study period. The patients enrolled in this study will receive standard-of-care supportive measures and all other medically necessary interventions as needed.

### Ethics

#### Research ethics approval

This study is performed in accordance with the Declaration of Helsinki. The protocol has been approved by the Wakayama Medical University Hospital Ethics Committee (approval number 2128). The trial protocol has also been registered in the protocol registration system at ClinicalTrials.gov (NCT03317886) and the University Hospital Medical Information Network Clinical Trials Registry (UMIN000029615). All patients will be scheduled only after comprehensive information concerning the nature, scope, and possible consequences of the clinical trial has been provided to them in an understandable way by the investigator. Written informed consent for the study will be obtained from each patient before the operation. The procedure, benefits, risks, and data management of this study will be clarified in detail for the patients during the preoperative conversation.

#### Dissemination policy

The results of the MAPLE-PD trial will be submitted to a peer-reviewed journal and will be presented at national and international conferences regardless of the trial outcomes. Authorship will be agreed in accordance with the MAPLE-PD trial publication policy and in line with international guidelines.

## Discussion

The use of the term *artery-first approach* during PD for PDAC has spread worldwide, and several case series have reported its feasibility [7, 18, 19]. The aims of the mesenteric approach, which is an artery-first approach, are early confirmation of resectability during operation and decrease of intraoperative blood loss and frequency of transfusion by early ligation of the IPDA. Further aims include increase of R0 rate by complete clearance of the connective tissues around the SMA and the SMV, including lymph nodes and plPh-II, which is the most favorable positive margin site for PDAC, and avoidance of cancer cell spread during operation, based on the concept of the nontouch isolation procedure [9, 10]. There is little evidence, however, of the oncological benefits of an artery-first approach, including the mesenteric approach for PDAC.

We therefore retrospectively compared the surgical outcomes between the conventional approach and the mesenteric approach to evaluate the oncological and surgical benefits of the mesenteric approach for PDAC [6]. We reported first that intraoperative blood loss was lower in the mesenteric approach in both resectable PDAC and BR PDAC, and the R0 rate was higher and the OS was better for the mesenteric approach in resectable PDAC, than in the conventional approach [6]. There is insufficient evidence to confirm our results because it was a retrospective, single-center study. The MAPLE-PD trial, which is a multicenter, prospective, randomized, controlled study therefore investigates whether the mesenteric approach can prolong survival for patients with PDAC who undergo PD compared with the conventional approach.

In the MAPLE-PD trial, the study population includes resectable PDAC as well as BR-PV PDAC cases, according to the 2017 NCCN definition. Some BR-PV PDAC cases might cross over into the mesenteric approach because surgeons want to rule out infiltration of the SMA via this approach. However, in the three consensus meetings for the MAPLE-PD trial, all surgeons participating in this study agreed with the inclusion of BR-PV PDAC in the trial. There are two reasons for this inclusion. First, in BR-PV PDAC cases without infiltration of the tissues around the SMA based on preoperative image findings, we rarely give up the surgical resection during the operation, so we do not consider it essential for BR-PV PDAC to confirm the resectability at the first step of the operation. Second, we strongly want to evaluate the oncological benefits of the mesenteric approach not only for resectable PDAC but also for BR-PV PDAC.

In the MAPLE-PD trial, resectability status is defined on the basis of 2017 NCCN criteria. Recently, a new international consensus on the definition and criteria of BR PDAC has been reported [20], including biological status and patient condition, in addition to the anatomical definition. Patients eligible for enrollment in the MAPLE-PD trial only have a performance status of 0–1 to compare purely surgical outcomes between the mesenteric and conventional approaches. However, biological status using serum CA 19-9 levels might be an important factor associated with survival for patients with PDAC; we would therefore like to compare the surgical outcomes between the two groups in the subgroup analysis based on these new biological criteria for BR PDAC [20].

If the MAPLE-PD trial shows that the mesenteric approach can improve survival by increasing R0 rate and/or avoiding spread of cancer cells during operation compared with the conventional approach, the mesenteric approach is expected to become a standard procedure during PD for PDAC worldwide, and survival of patients with PDAC in the future may improve.

## Trial status

The MAPLE-PD trial was opened in January 2018. At the time of the submission of this paper (October 2018), the protocol version is version 1-5, twelve institutions were actively recruiting, and three institutions were pending. The completion date is estimated to be December 2021.

## Additional file

Additional file 1: Institution list. (DOC 122 kb)

## Abbreviations

BR-PV PDAC: borderline resectable pancreatic ductal adenocarcinoma with portal vein invasion
DGE: Delayed gastric emptying
EDC: Electronic data capture
IPDA: Inferior pancreaticoduodenal artery
NCCN: National Comprehensive Cancer Network
OS: Overall survival
PD: Pancreaticoduodenectomy
PDAC: Pancreatic ductal adenocarcinoma
plPh-II: Pancreatic head plexus II
POPF: Postoperative pancreatic fistula
PV: Portal vein
SMA: Superior mesenteric artery
SMV: Superior mesenteric vein

## References

[1] Strobel O, Hank T, Hinz U, Bergmann F, Schneider L, Springfeld C (2017). Pancreatic cancer surgery: the new R-status counts. Ann Surg.

[2] Jamieson NB, Foulis AK, Oien KA, Going JJ, Glen P, Dickson EJ (2010). Positive mobilization margins alone do not influence survival following pancreaticoduodenectomy for pancreatic ductal adenocarcinoma. Ann Surg.

[3] Büller MW, Werner J, Weitz J (2010). R0 in pancreatic cancer surgery: surgery, pathology, biology, or definition matters?. Ann Surg.

[4] Verbeke CS, Leitch D, Menon KV, McMahon MJ, Guillou PJ, Anthoney A (2006). Redefining the R1 resection in pancreatic cancer. Br J Surg.

[5] Japanese Pancreas Society (2016). General Rules for the Study of Pancreatic Cancer.

[6] Hirono S, Kawai M, Okada K, Miyazawa M, Shimizu A, Kitahata Y (2017). Mesenteric approach during pancreaticoduodenectomy for pancreatic ductal adenocarcinoma. Ann Gastroenterol Surg.

[7] Weitz J, Rahbari N, Koch M, Büchler MW (2010). The “artery first” approach for resection of pancreatic head cancer. J Am Coll Surg.

[8] Sanjay P, Takaori K, Govil S, Shrikhande SV, Windsor JA (2012). “Artery-first” approaches to pancreatoduodenectomy. Br J Surg.

[9] Nakao A, Takagi H (1993). Isolated pancreatectomy for pancreatic head carcinoma using catheter bypass of the portal vein. Hepatogastroenterology.

[10] Nakao A (2016). The mesenteric approach in pancreatoduodenectomy. Dig Surg.

[11] Bassi C, Marchegiani G, Dervenis C, Sarr M, Abu Hilal M, Adham M (2017). The 2016 update of the International Study Group (ISGPS) definition and grading of postoperative pancreatic fistula: 11 years after. Surgery.

[12] Wente MN, Bassi C, Dervenis C, Fingerhut A, Gouma DJ, Izbicki JR (2007). Delayed gastric emptying (DGE) after pancreatic surgery: a suggested definition by the International Study Group of Pancreatic Surgery (ISGPS). Surgery.

[13] Wente MN, Veit JA, Bassi C, Dervenis C, Fingerhut A, Gouma DJ (2017). Postpancreatectomy hemorrhage (PPH): an International Study group of Pancreatic Surgery (ISGPS) definition. Surgery.

[14] Dindo D, Demartines N, Clavien PA (2004). Classification of surgical complications: a new proposal with evaluation in a cohort of 6336 patients and results of a survey. Ann Surg.

[15] Tempero MA, Malafa MP, AI-Hawary M, Asbun H, Bain A, Beehrman SW, et al. Pancreatic adenocarcinoma, version 2.2017. JNCCN. 2017;15:1028–61. 10.6004/jnccn.2017.0131 28784865

[16] Hirono S, Yamaue H (2015). Tips and tricks of the surgical technique for borderline resectable pancreatic cancer: mesenteric approach and modified distal pancreatectomy with en-bloc celiac axis resection. J Hepatobiliary Pancreat Sci.

[17] Hirono S, Kawai M, Tani M, Okada K, Miyazawa M, Shimizu A (2014). Indication for the use of an interposed graft during portal vein and/or superior mesenteric vein reconstruction in pancreatic resection based on perioperative outcomes. Langenbecks Arch Surg.

[18] Inoue Y, Saiura A, Yoshioka R, Ono Y, Takahashi M, Arita J (2015). Pancreatoduodenectomy with systematic mesopancreas dissection using a supracolic anterior artery-first approach. Ann Surg.

[19] Kawabata Y, Tanaka T, Nishi T, Monma H, Yano S, Tajima Y (2012). Appraisal of a total meso-pancreatoduodenum excision with pancreaticoduodenectomy for pancreatic head carcinoma. Eur J Surg Oncol.

[20] Isaji S, Mizuno S, Windsor JA, Bassi C, Castillo CF, Hackert T (2018). International consensus on definition and criteria of borderline resectable pancreatic ductal adenocarcinoma 2017. Pancreatology.

